# Supplementary material for: Heterologous Expression of Nitrate Assimilation Related-Protein DsNAR2.1/NRT3.1 Affects Uptake of Nitrate and Ammonium in Nitrogen-Starved Arabidopsis
Source: Int J Mol Sci. 2020 Jun 4;21(11):4027. doi: 10.3390/ijms21114027 (PMC7312895; doi:10.3390/ijms21114027)

Table S1. List of PCR primers [a].

Primer Name	Sequence (5′-3′)
DsNRT3.1-F	ATGGCGGTGCGAGGATTAAC
DsNRT3.1-R	CCAAATTAAGTAGCTAGCTTCGAC
121DsNRT3.1(X)-F	TCTAGAATGGCGGTGCGAGGATTAAC
121DsNRT3.1(S)-R	GAGCTCGTAGCTAGCTTCGACTTCTTC
121DsNRT3.1(K)-R	GGTACCGTAGCTAGCTTCGACTTCTTC
AtActin1-F	GAAAATGGCTGATGGTGAAG
AtActin1-R	CTCATAGATAGGAACAGTGTGGC
DsNRT3.1-qF	ACTCCAAAGTCGCCCTCAAA
DsNRT3.1-qR	AGCGGCGTATGGTTTAGTCG
DsActin7-qF	CGGTGGCTCTATCCTCGCTT
DsActin7-qR	TTCCTGTGGACGATTGACGG
AtActin2-qF	GGTAACATTGTGCTCAGTGGTGG
AtActin2-qR	AACGACCTTAATCTTCATGCTGC
AtNRT2.1-qF	CGGTGGACACAGAGCATAAGG
AtNRT2.1-qR	GGATGATAGGGACAAGTGGTGC
AtNRT2.2-qF	GCCGACCAACACAAAGAAAA
AtNRT2.2-qR	CAACTCCAGCGTTTCCAATG
AtNRT2.3-qF	GCATTCTCGTCCTCAGTCTCG
AtNRT2.3-qR	TCTTCACAGCAAACCAGAAAACC
AtNRT2.4-qF	GGACACCGAACACAAAGCCA
AtNRT2.4-qR	CAAGAGGAGCAGCAGCGAAA
AtNRT2.5-qF	CTCTCTCTGCTTTCGCCGTT
AtNRT2.5-qR	TTCCCCACATCATCTTTCTCC
AtNRT2.6-qF	CGACCTGACCAAAACCGACA
AtNRT2.6-qR	TGTAAGCATAAGTGAGAAGGCGG
AtNRT2.7-qF	ATCTCAACGCAACACCAAACC
AtNRT2.7-qR	TGAAAGGCTCGTGAGTGTGG
AtNRT3.1-qF	CGACGATGCCAAGAAAACCA
AtNRT3.1-qR	CCACGACGGAGAAGACACTGAA

[a] The specificity and efficiency of the primers were confirmed by the amplification curve, melt curve and melt peak figures, which are shown below.

DsNRT3.1

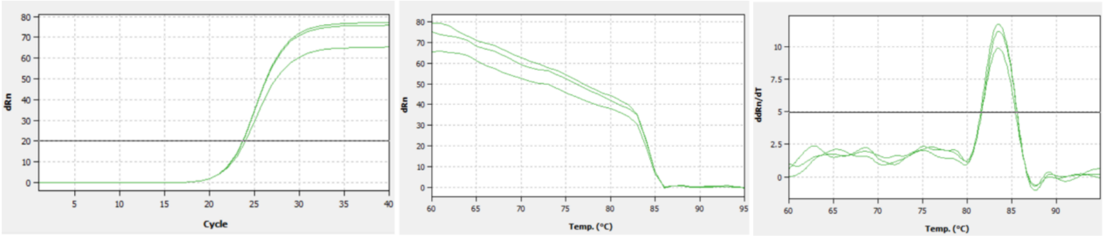

DsActin7

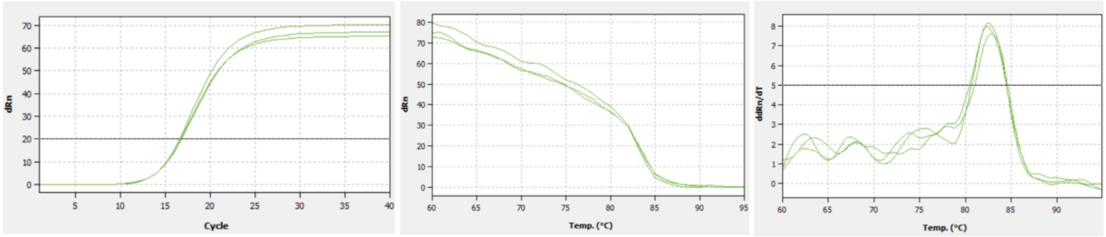

AtNRT3.1

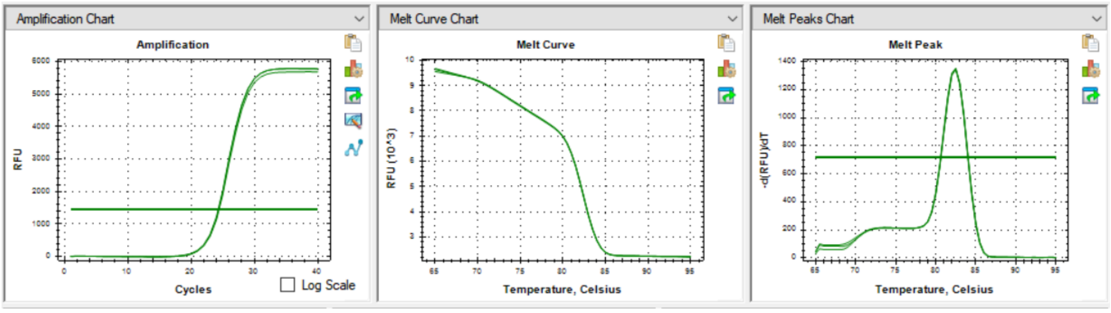

AtNRT2.1

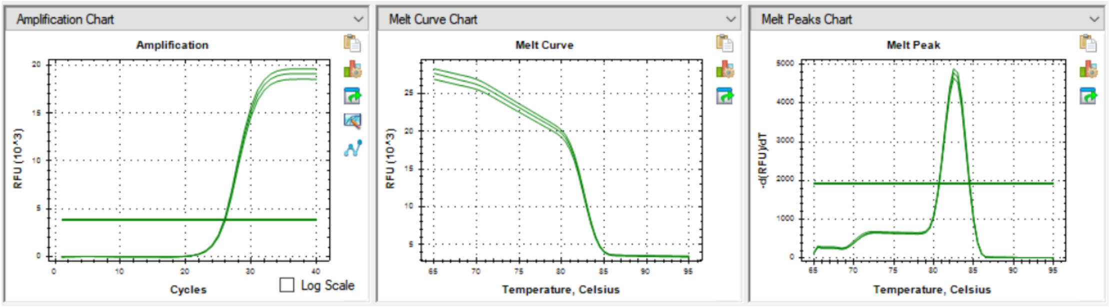

AtNRT2.2

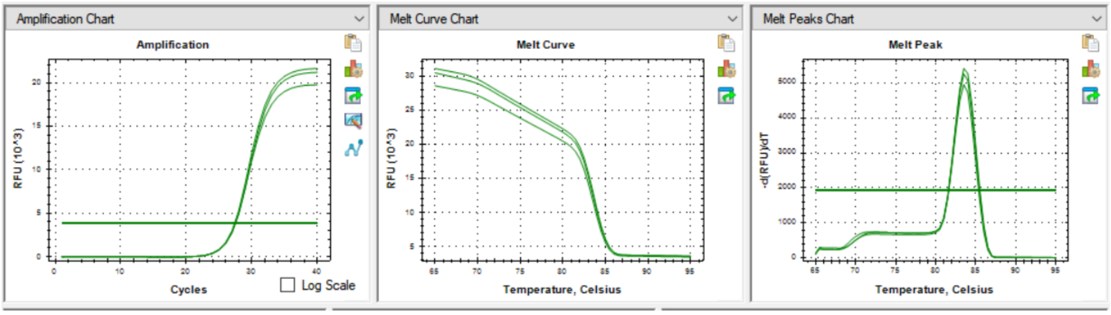

AtNRT2.3

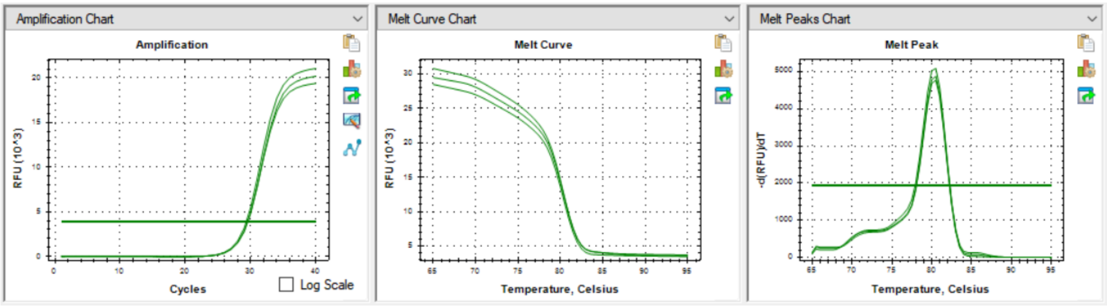

AtNRT2.4

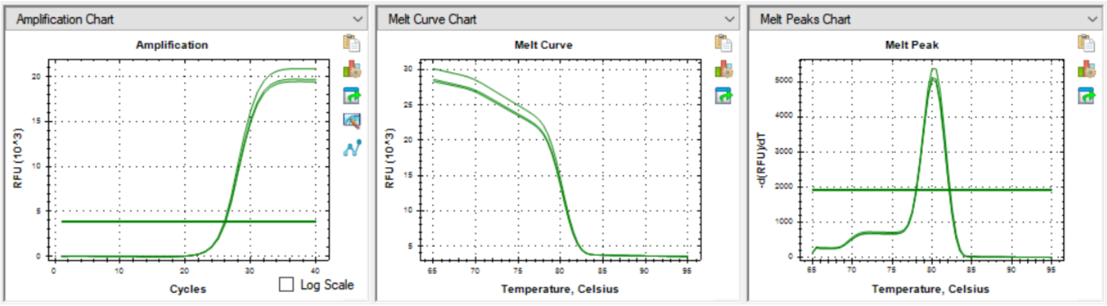

AtNRT2.5

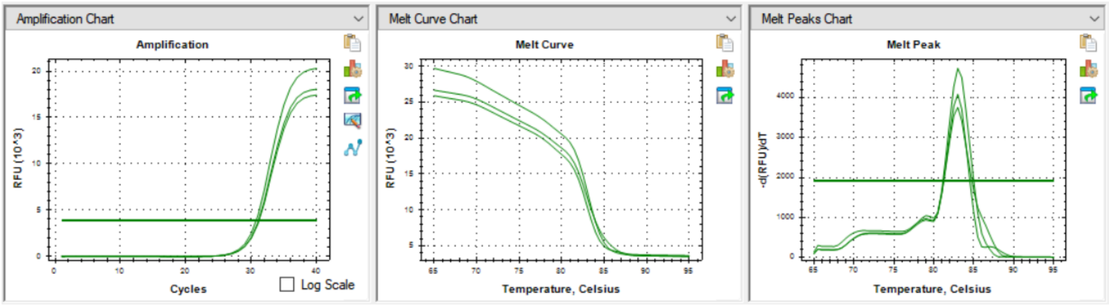

AtNRT2.6

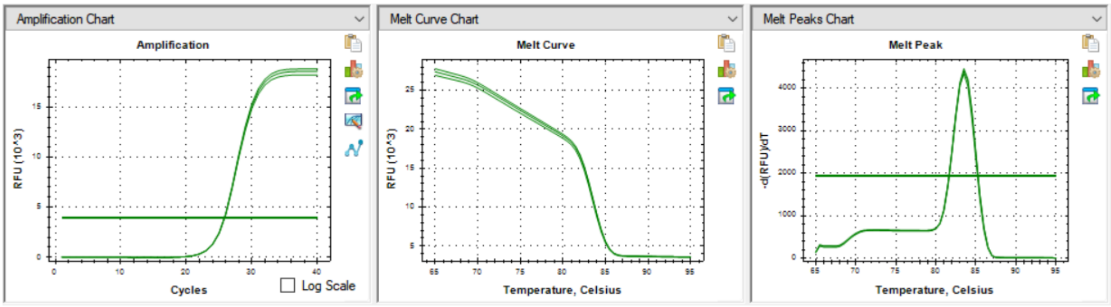

AtNRT2.7

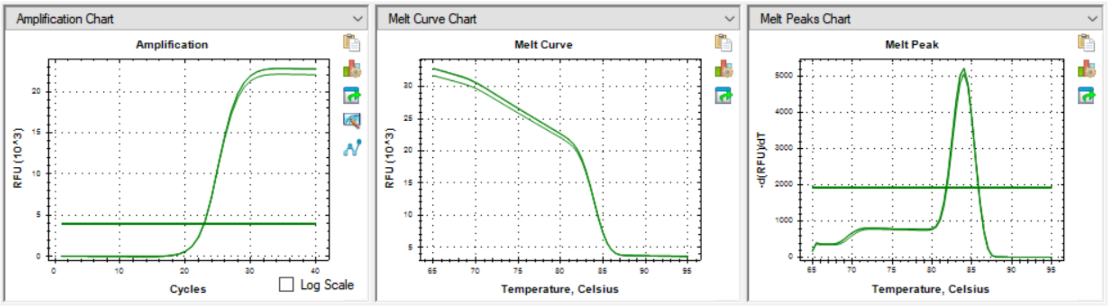

AtActin2

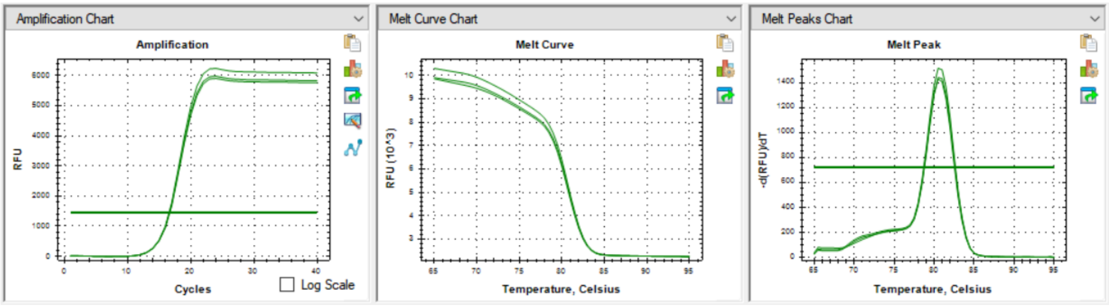

Supplement: Supplementary file 1 [file ijms-21-04027-s001.pdf]
